# Effect of video-based interventions on emergence delirium in pediatric patients: a systematic review and meta-analysis of randomized controlled trials

**DOI:** 10.1016/j.jped.2024.06.016

**Published:** 2024-09-06

**Authors:** Yue Wang, Lifang Wang, Nan Liang, Kan Wang

**Affiliations:** China-Japan Friendship Hospital, Department of Anesthesiology, Beijing, China

**Keywords:** Pediatric, Pediatric anesthesia, Emergence delirium, Audiovisual aids, Neurocognitive disorders

## Abstract

**Objective:**

Emergence delirium is frequently observed in pediatric patients. With advancements in video-based interventions, such as cartoons, video games, and virtual reality, these modalities may contribute to a reduced incidence of emergency delirium among children. However, robust evidence supporting their efficacy remains necessary.

**Methods:**

The authors conducted a systematic search across multiple databases, including Embase, MEDLINE, and Cochrane Library, to identify all randomized controlled trials comparing video-based interventions with control treatments in pediatric emergence delirium. Data were aggregated and analyzed using Review Manager 5.4 to evaluate the effectiveness of video-based interventions.

**Results:**

The analysis included eight randomized controlled trials comprising 872 children. The intervention group showed a trend toward lower Pediatric Anesthesia Emergence Delirium scores (*p* = 0.10) and fewer emergence delirium events (*p* = 0.52). Seven studies demonstrated that video-based interventions significantly reduced preoperative anxiety, as indicated by decreased scores on the modified Yale Pre-operative Anxiety Scale (*p* < 0.00001). Anesthesia duration did not significantly differ between the intervention and control groups (*p* = 0.16). Notably, subgroup analyses revealed a significant reduction in Pediatric Anesthesia Emergence Delirium scores among children under seven years of age (*p* = 0.001).

**Conclusions:**

Video-based interventions were linked to lower Pediatric Anesthesia Emergence Delirium scores and a decreased incidence of emergence delirium events. However, these results did not reach statistical significance across the broader sample. Notably, in children under seven, these interventions significantly reduced the scores.

**Level of evidence:**

III

## Introduction

Emergence delirium (ED) represents a significant concern for anesthesiologists and nurses managing pediatric patients during the postoperative recovery period. Typically manifesting in the recovery room and can last between 10 and 45 min after general anesthesia,^1^, ^2^, ^3^ ED in pediatric patients can be as high as 80 %, compared to 5–10 % in adults.^4^^,^^5^ It is characterized by persistent cognitive disturbances like uncooperativeness, irritability, and inconsolability, often accompanied by crying and physical agitation.^6^ Although ED typically manifests as a transient challenge during immediate postoperative care, studies indicate that its effects may extend beyond the recovery phase. Research has shown that elevated preoperative anxiety levels are associated with a higher incidence of ED and can lead to prolonged anxiety and sleep disturbances that persist for up to two weeks after surgery.^7^

While the specific mechanisms underlying ED are not fully understood, several risk factors have been identified, including the use of volatile anesthetics,^4^^,^^8^ patient age,^9^ preoperative anxiety,^7^^,^^10^ and interactions with healthcare providers.^11^^,^^12^ Considering these risk factors, current strategies aimed at reducing the incidence of ED primarily involve both pharmacological and non-pharmacological approaches. Substances such as propofol, fentanyl, dexmedetomidine, and low-dose ketamine have been found to effectively reduce the occurrence of ED.^13^, ^14^, ^15^, ^16^ Beyond pharmacological interventions, non-pharmacological treatments, specifically behavioral management techniques, represent a viable therapeutic option.^6^

Despite ongoing research, there remains a lack of consensus on the efficacy of visualization techniques such as virtual reality (VR) in preventing ED. Some studies, such as those by Ryu et al., suggest that VR can reduce preoperative anxiety without significantly impacting the occurrence or severity of ED.^17^ Conversely, other research indicates that interventions like tablet computers can lessen anxiety during anesthesia induction in younger children but fail to significantly affect ED outcomes.^18^ Therefore, this systematic review aims to explore the impact of preoperative video-based interventions on ED in pediatric patients, seeking to clarify their potential in both causing and preventing this challenging condition.

## Materials and methods

The current study including eight RCTs was conducted in line with the principles of the Preferred Reporting Items for Systematic Reviews and Meta‐ Analyses (PRISMA) guidelines and registered on PROSPERO (registration ID: CRD42023488948).

### Search strategy and study selection

Multiple databases comprising Embase, MEDLINE, and Cochrane Library were thoroughly searched for all RCTs from inception to January 31, 2024, by two independent researchers (Y. W. and LF. W). The search terms included visualization and emergence delirium. Details of the search strategy are in Supplemental Appendix 1. Additionally, reviews, meta-analyses, and reference lists of all retrieved reports were also searched for additional eligible studies not identified in the initial electronic database search. The language was restricted to English. Any controversy about article selection or data abstraction was resolved through consensus with the third researcher (K. W.).

### Inclusion and exclusion criteria

Studies were included if the PICOS (population, intervention, comparison, outcomes, and study design) guidelines were met: (a) Population: pediatric patients (< 18 years old) undergoing general anesthesia; (b) Intervention: perioperative use of any kind of visualization (virtual reality exposure, cartoon video, tablet game, etc.); (c) Comparison: no visualization intervention; (d) Outcomes: PAED scores, the incidence of ED, and preoperative anxiety; (e) Study design: RCTs.

The exclusion criteria were as follows: (a) visualization intervention combined with drugs or voice as an intervention group; (b) data were not able to meta-analysis; (c) unpublished materials or articles published in the form of summaries, reviews, case reports, letters and protocols.

### Data extraction

From each included study, predefined data were extracted: the first author, publication year, country, study grouping, number of patients, age, American Society of Anesthesiologists Physical Status (ASA PS) of surgeries, general anesthetics, surgical and anesthesia time, intervention period, type of visualization, the incidence of events or means and standard deviations of the outcome data, and the methods and criteria for the outcome data measurements. Two researchers (Y. W. and LF. W) independently conducted the process of data extraction, and a third researcher (K. W.) verified the final result.

### Quality assessment

Two researchers (Y. W. and K. W.) independently assessed the methodological integrity of the studies using the Cochrane risk of bias assessment tool. This tool encompasses various factors, including random sequence generation, allocation concealment, blinding of staff, participants, and outcome evaluators, completeness of outcome data, selective outcome reporting, and additional biases. Studies were then classified based on their adherence to these quality criteria: low risk (full compliance with all criteria), moderate risk (partial or unclear adherence to one or more criteria), or high risk (failure to meet or absence of one or more criteria).

### Statistical analysis

Review Manager 5.4 was used for the analyses of results. For continuous outcomes with identical measurement units, the mean difference (MD) along with a 95 % confidence interval (CI) was computed. Dichotomous outcomes were assessed using the relative risk (RR) and its 95 % CI. Forest plots were employed to display these outcomes in a graphical format. A P-value < 0.05 was deemed to indicate statistical significance for the overall effect size. The I^2^ value and the Mantel-Haenszel method were used to evaluate heterogeneity among studies. An I^2^ value of 50 % or higher suggested substantial heterogeneity, prompting the use of random-effects models. Subgroup analyses were anticipated to identify potential sources of heterogeneity. In cases where heterogeneity was found to be minimal, fixed-effect models were applied.

## Results

### Study identification and selection

**Figure 1:**
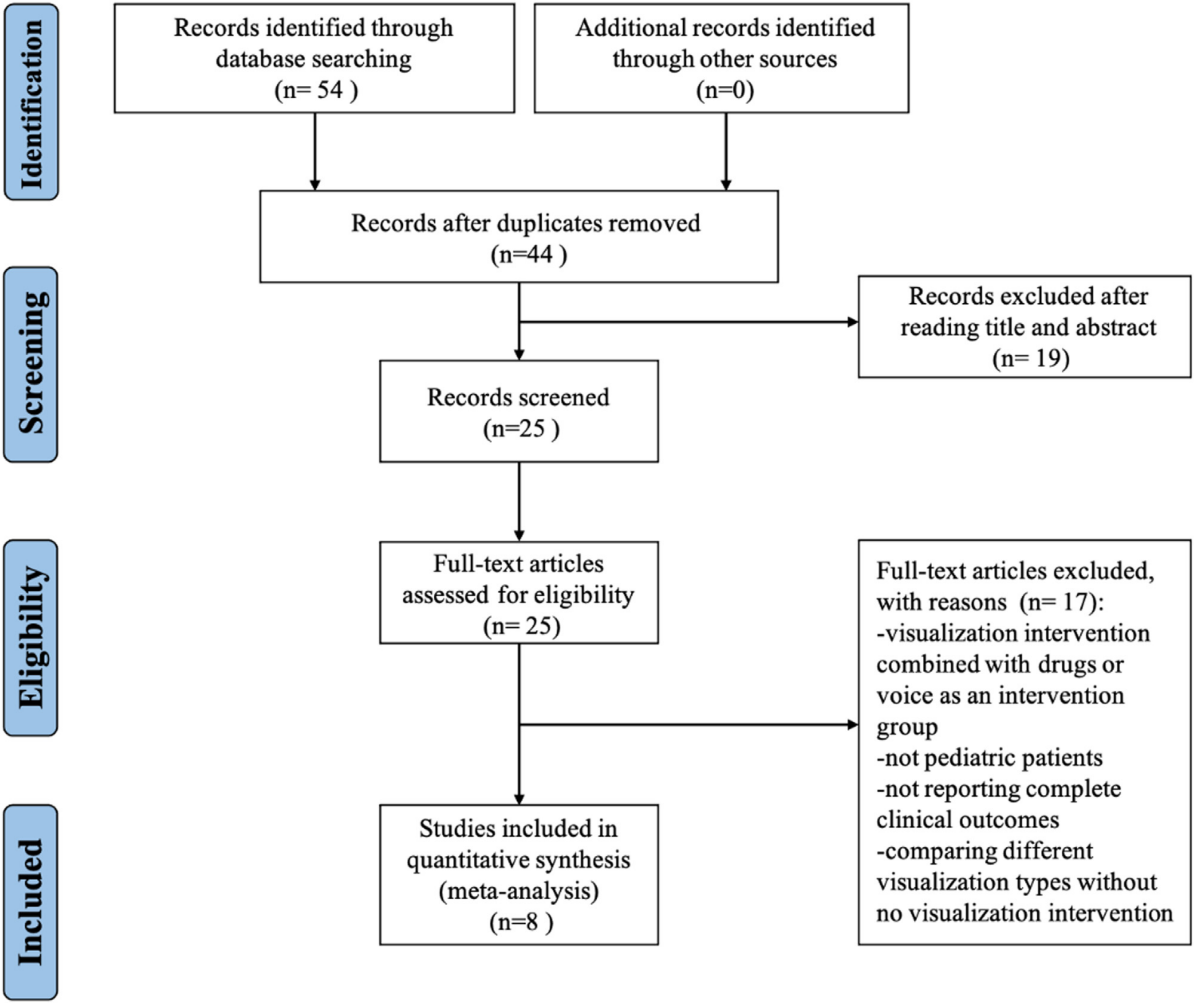
Flow diagram of the search strategy for systematic review and meta-analysis.

The initial search identified 54 potentially relevant studies. After removing 10 duplicates, 19 studies were excluded following a preliminary review of titles and abstracts. This process left 25 studies for a detailed full-text assessment. Of these, 17 were found unsuitable for inclusion, leading to the incorporation of 8 studies into the meta-analysis, as depicted in Figure 1.

### Study characteristics

Table 1 outlines the fundamental attributes of the studies included in this analysis. These studies were published between 2015 and 2023, encompassing a demographic of children aged between 4 and 8.05 years. The interventions varied, with three studies examining the effects of VR exposure versus no VR intervention, three studies comparing cartoon videos with no video intervention, one study assessing the impact of tablet games versus no game, and one study evaluating video alongside parental presence versus parental presence alone. All involved surgeries were classified as ASA Physical Status I or II. The types of general anesthetics used included sevoflurane, desflurane, and N_2_O, with some cases using remifentanil or no remifentanil. Emergence delirium was quantitatively assessed using the Pediatric Anesthesia Emergence Delirium (PAED) score,^9^ and pre-operative anxiety was measured with the modified Yale Preoperative Anxiety Scale (mYPAS).^19^

**Table 1:** Characteristics of the included randomized-controlled trials.

Author and year	Country	Grouping	N	Age (years)	Visualization type	Intervention period	General anesthetics	ASA PS (I/II)	Surgical time (min)	Anesthesia time (min)	Emergence delirium assessment	Preoperative Anxiety
Kim 2015	South Korea	video	34	5.5 ± 1.0	animated cartoon video in a smartphone	throughout induction of anesthesia	sevoflurane+ N2O+ remifentanil	34/0	37.0 ± 16.5	60.0 ± 19.3	PAED scale	mYPAS scores
		parental presence	33	5.3 ± 1.4				32/1	40.9 ± 10.1	62.9 ± 14.5		
		video+ parental presence	37	5.0 ± 1.3				37/0	39.1 ± 10.6	61.7 ± 11.5		
Wu 2022	China	VR exposure	48	7.35±2.29	a VR video showing a realistic interactive immersive virtual version of the perioperative process	during pre-anesthesia visit	sevoflurane± remifentanil	45/6	/	/	PAED scale	mYPAS scores
		control	48	7.56 ± 3.63				45/3				
Dwairej 2019	Jordan	intervention	64	6.61±1.72	combined interactive video distraction through the handheld video game	throughout induction of anesthesia	/	/	/	/	PAED scale	mYPAS scores
		control	64	6.50±1.86								
Ryu 2019	Korea	VR	41	6 ± 1.54	a VR video introduced and explained the perioperative preparation process	1 h prior to entering the operating theater	sevoflurane/ desflurane	39/2	20 ± 15.36	43.55 ± 23.05	PAED scale	mYPAS scores
		control	39	6.36±2.31				37/2	25.33 ± 19.24	46.78 ± 26.94		
Tang 2023	China	cartoon video	40	5.75±1.21	a cartoon video of the child’ s own choice	throughout waiting, anesthesia induction and recovery periods	sevoflurane+ remifentanil	/	34.45±12.07	47.35±15.16	PAED scale	mYPAS scores
		control	40	5.68±1.58					34.63±12.20	46.23±14.09		
Eijlers 2019	Netherlands	virtual reality exposure	73	8.05±3.40	a VR video showing a realistic interactive immersive virtual version of the perioperative process	during waiting period	sevoflurane	72/22	/	/	PAED scale	mYPAS scores
		care as usual	97	7.96±3.84				65/32				
Clausen 2021	Denmark	tablet game	30	4.4 ± 0.3	play a game on a tablet computer	in the holding area before anesthesia	sevoflurane ± fentanyl/ remifentanil	/	16.7 ± 2.2	37.8 ± 3.7	PAED scale	mYPAS scores
		control	30	4.4 ± 0.2					15.2 ± 2.1	39.1 ± 3.5		
Zhang 2022	China	video training	77	5	during the pre-operative visit	watching video to receive breathing training	sevoflurane+ remifentanil	72/5	43	70	PAED scale	mYPAS scores
		control	77	5				71/6	45	75		

### Risk of bias assessment

A methodological quality assessment was conducted, and the results are shown in Supplemental Figure 1.

### Primary outcomes

**Figure 2:**
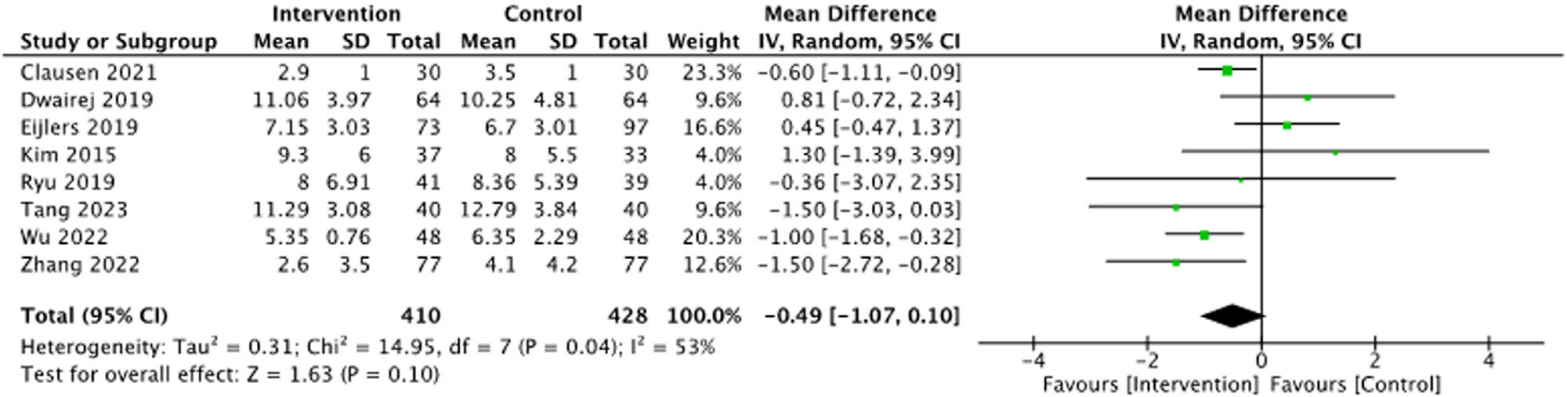
Forest plot for the effect of visualization on PAED score.

All studies involving 872 children provided data on PAED scores. All studies used the PAED scale for estimating ED. Compared with the control, children in the visualization intervention group tended to have lower PAED scores (Figure 2, *p* = 0.10).

### Secondary outcomes

**Figure 3:**
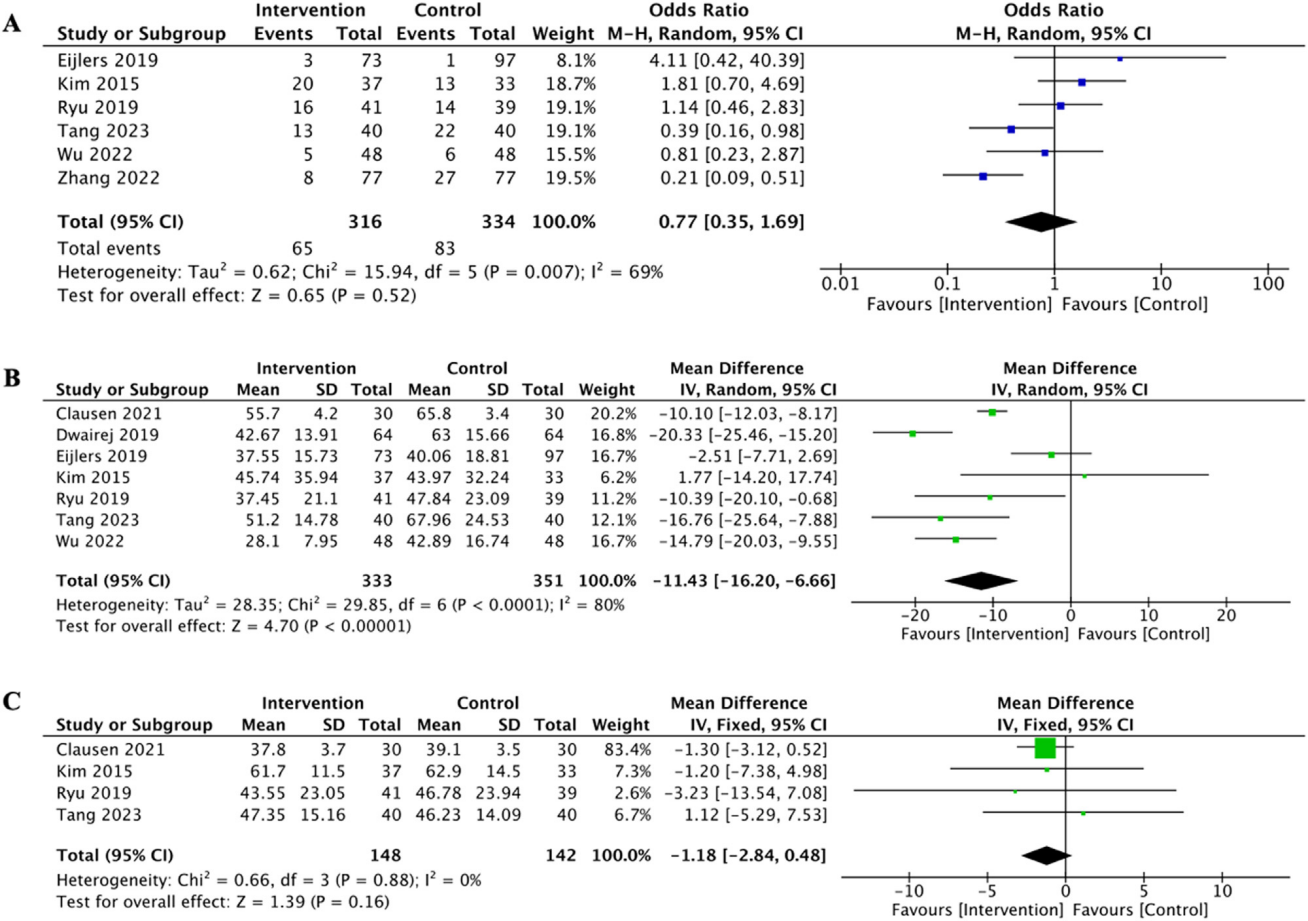
Forest plot for secondary outcomes. A. Forest plot for the effect of visualization on ED. B. Forest plot for the effect of visualization on mYPAS scores. C. Forest plot for the anesthesia time.

Data on ED events were provided by six studies involving 650 children. The analysis revealed no significant difference in the incidence of ED events between the intervention and control groups (Figure 3A, *p* = 0.52).

Additionally, seven studies reported data on the mYPAS scores. The results indicated that visualization interventions significantly reduced mYPAS scores, suggesting that these interventions effectively decrease preoperative anxiety in children (Figure 3B, *p* < 0.00001). Moreover, the meta-analysis assessed differences in anesthesia duration between the intervention and control groups finding no significant variation (Figure 3C, *p* = 0.16).

### Subgroup analyses

To accommodate the diversity in age among the pediatric participants, the authors divided the data into two separate age groups: children under 7 old and children 7 years and above. Each subgroup comprised four trials. The analysis demonstrated a notable decrease in PAED scores in the younger subgroup, suggesting that visualization interventions were especially beneficial for children under the age of 7 (Figure 41, *p* = 0.001).

**Figure 4:**
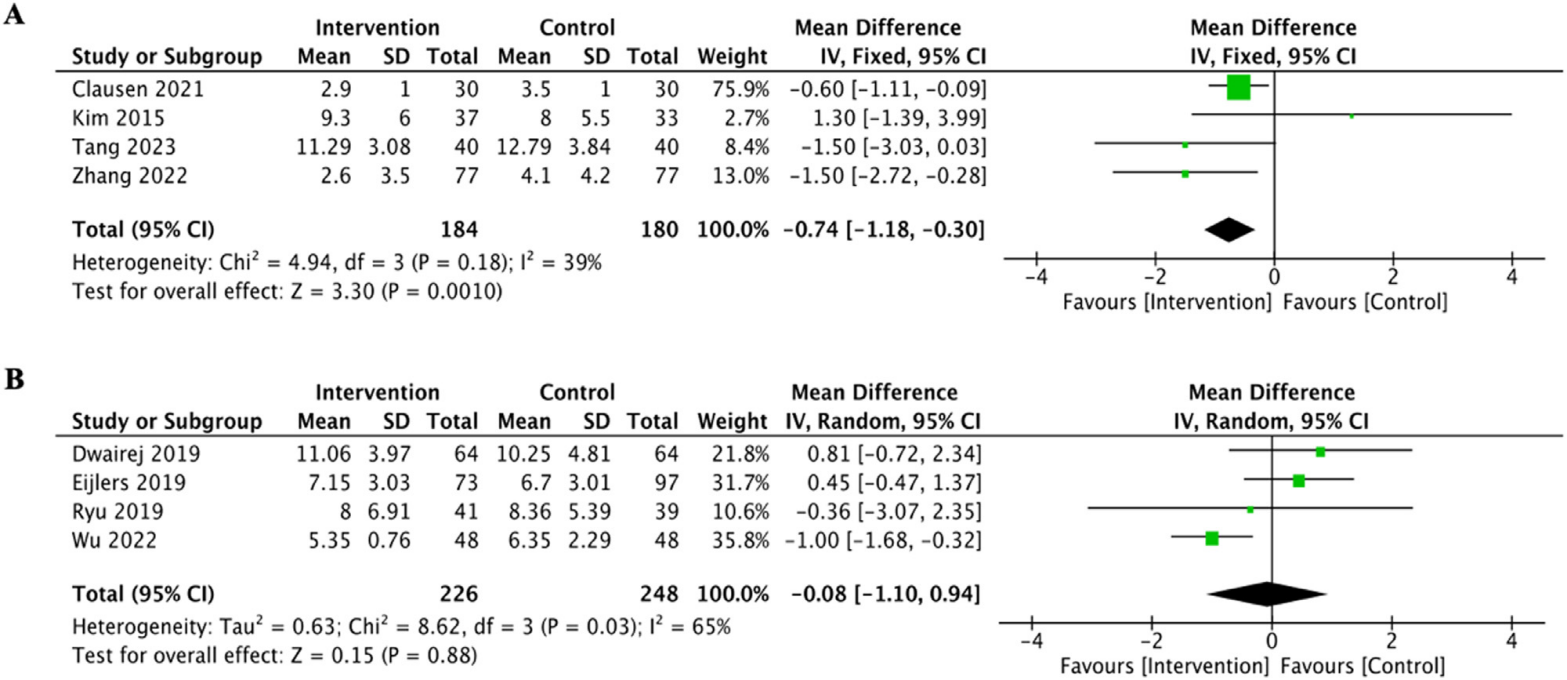
Forest plot for the effect of visualization on PAED score according to age. A. age <7 years old. B. age ≥ 7 years old.

## Discussion

In this comprehensive systematic review and meta-analysis, the authors evaluated the effectiveness of video-based interventions in reducing postoperative ED among pediatric patients. The present results demonstrated a consistent trend towards lower PAED scores, indicating a potential beneficial effect of video-based interventions in alleviating ED. While the overall reductions in ED rates did not reach statistical significance, the data revealed a significant decrease in PAED scores specifically in children under the age of 7. This finding underscores the effectiveness of video-based interventions in this particular age group.

In the analysis of PAED scores, the authors noted an initial overall heterogeneity of 53 %. This was notably influenced by the studies of Dwairej 2019^20^ and Eijlers 2019,^21^ which contributed significantly to this variability. Upon exclusion of these studies, heterogeneity markedly decreased to 8 %, with the subsequent analyses showing a significant reduction in PAED scores following video intervention (*p* < 0.01). Several factors may account for the observed reduction in heterogeneity. Age variation: The participants in Dwairej 2019 ranged from 5 to 11 years, while those in Eijlers 2019 were between 4 and 12 years old. The variation in age suggests that different age groups may respond differently to interventions,^22^ highlighting the importance of considering age when assessing heterogeneity and interpreting results. Subjectivity in PAED scoring: The PAED scale, while useful, is subject to subjective interpretation, which can introduce scoring bias.^23^ This highlights the importance of acknowledging the potential influence of subjective assessments on study outcomes. Diversity in intervention methods: The inclusion of both video and virtual reality (VR) interventions highlights how different intervention modalities might influence results, suggesting that the specifics of the intervention content are critical and merit further exploration. Sample size concerns: The small sample sizes in five of the studies, each with fewer than 50 participants, likely contributed to the heterogeneity. This highlights the necessity for larger-scale studies to improve the robustness of findings. Future research should address these factors by focusing on diverse age groups, increasing sample sizes, and providing a more detailed analysis of how different video interventions impact delirium assessment. While the reduced variability aids in understanding the effects of video interventions, caution is advised in interpreting these results, and further validation in subsequent studies is essential.

The age-stratified analysis revealed a significant reduction in PAED scores among children under 7 years old, accompanied by reduced heterogeneity in this subgroup. This suggests that video-based interventions are particularly effective in younger children, supporting their use to decrease the incidence of ED in this age group. However, in children older than 7 years, the interventions did not show a significant effect, highlighting potential individual differences and the influence of age on intervention efficacy. Additionally, video-based interventions were effective in reducing preoperative anxiety scores, consistent with findings from previous research.^24^^,^^25^ The present analysis also indicated that these interventions do not significantly extend anesthesia time, suggesting that they can mitigate delirium without adversely affecting the duration of anesthesia or the anesthesia procedures themselves. In conclusion, while this study observed promising trends in the effectiveness of video-based interventions, it is crucial to approach these results with caution due to inherent study limitations. Future research should further explore the age-specific responses to these interventions to optimize their use in clinical settings.

In the meta-analysis investigating the incidence of ED, the authors initially observed substantial heterogeneity, measured at 69 %. This was mainly attributed to differences in the conducted by Zhang LN 2022^26^ and Tang 2023.^27^ After excluding these studies, the heterogeneity dropped to 0 %, indicating minimal variation among the remaining studies. Despite this adjustment, the results showed that video interventions did not significantly reduce the overall incidence of ED. Notably, the study by Zhang LN 2022 involved educating children on breathing movements postoperatively through videos, whereas Tang 2023 allowed children to select their favorite animation to watch for 30 min before surgery. Both studies utilized conventional videos instead of VR technologies. Intriguingly, upon analyzing these two studies, a significant reduction in ED occurrence was observed (*p* < 0.01). This indicates that the educational value of the video content or the choice given to children may have a positive impact on delirium outcomes. This finding aligns with observed trends in adult populations, where video interventions have similarly reduced delirium incidence (*p* = 0.044).^28^ Such trends underscore the potential impact of intervention content and its customization on outcomes, suggesting that similar approaches might be beneficial for pediatric patients as well. In conclusion, the results underscore the importance of considering the specific details and design of the intervention content when interpreting outcomes related to ED rates. The significant impact of these factors warrants further exploration in future research to enhance understanding and optimize the use of video interventions in clinical settings.

Emergence delirium significantly complicates the recovery process after pediatric surgeries, prompting a shift towards non-pharmacological strategies for its mitigation. Video-based interventions have risen to prominence within this context, offering unique benefits. Despite their increasing use, the precise mechanisms through which these interventions mitigate postoperative delirium remain elusive. Existing research highlights a strong link between preoperative anxiety, postoperative pain, and the development of postoperative delirium.^7^^,^^10^ Video interventions have consistently demonstrated effectiveness in reducing preoperative anxiety and mitigating postoperative pain, suggesting they might similarly impact the incidence of ED.^25^^,^^29^ Evidence supporting the potential of video interventions to reduce ED is compelling. However, further empirical studies are required to fully establish these effects. This gap underscores the need for continued research to clarify the benefits and operationalize the most effective video-based strategies in pediatric surgical care.

Video interventions in pediatric care are primarily categorized into two types: videos shown on traditional screens and those experienced through VR devices. These interventions cover various content types such as animations, games, and educational materials.^30^ Unlike animations, which usually require passive viewing, both games and instructional actively engage children through interactive elements. This interactivity is thought to amplify the therapeutic effectiveness of the videos.^31^ Empirical evidence supports the superior effectiveness of interactive videos over traditional animations in reducing anxiety in children. Such findings the potential for these interactive modalities to also reduce ED. However, the evidence base remains incomplete, and further experimental research is necessary to conclusively determine the effectiveness of interactive video interventions in mitigating ED.

VR stands out as a cutting-edge technological innovation, offering immersive experiences that hold unique benefits in medical applications, particularly for alleviating anxiety and delirium. As a medium, VR provides an engaging way to deliver therapeutic content. However, its application in pediatric settings faces several notable challenges.

One primary concern is the discomfort that younger children often experience when using VR glasses, which are typically designed with adults in mind. This discomfort can lead to reluctance or outright refusal to use VR technologies. In the meta-analysis, studies by Eijlers et al. and Jung et al. highlight this issue, with reported refusal rates due to discomfort at 23 % and dropout rates at 5 %,^21^^,^^32^ respectively. Additionally, the limited availability of high-quality VR content tailored for children further complicates its use. Most VR platforms focus primarily on video playback, lacking interactive and child-friendly content that could enhance engagement and therapeutic outcomes. These challenges underscore the necessity for advancements in VR hardware and content development customized for the pediatric population. Improving the comfort of VR headsets and broadening the range of content specifically created for children are crucial steps toward maximizing the potential of VR to effectively reduce ED.^33^ These enhancements could substantially enhance the acceptance and efficacy of VR as a therapeutic tool in pediatric care.

The integration of video interventions with pharmacological treatments has shown promising results in reducing the incidence of delirium, although outcomes vary depending on the specific medications used. A study by Wu et al. demonstrated that the combination of dexmedetomidine with anesthesia significantly lowered ED rates (*p* = 0.001),^34^ illustrating the potential of synergistic effects between video interventions and certain sedatives. Conversely, the research conducted by Ahmadpour et al. found that combining midazolam with video interventions did not lead to a statistically significant reduction in ED incidence (*P* = 0.43).^35^ These mixed results highlight the influence of different pharmacological agents when used in conjunction with video-based strategies. The variability underscores the need for further detailed investigations into how various drug-video combinations affect ED outcomes. Understanding these dynamics is crucial for optimizing treatment protocols and can provide valuable insights into more effective strategies for managing delirium in clinical settings.

This study acknowledges several limitations that merit consideration. Primarily, the observed high heterogeneity can be attributed to factors such as diverse age groups, small sample sizes, variability in the timing of delirium assessments, and the inconsistent quality of original data. Additionally, while there is a discernible trend suggesting that video interventions may reduce the incidence of ED, the limited number of studies included in this analysis necessitates further investigation through additional randomized controlled trials to conclusively determine the effectiveness of these interventions. A particular concern is the unclear impact of video interventions on children older than 7 years. This ambiguity highlights the potential need to tailor video content more closely to the physiological and cognitive characteristics of school-age children. Future research should focus on developing and testing video-based interventions specifically designed to address the unique needs of this age group and effectively reduce PAED scores. The authors recommend that future studies take a more systematic and detailed approach to investigate the correlation between preoperative visual interventions and ED. This should include a comprehensive analysis of factors such as age-specific responses, timing of interventions, and the types of delirium assessments utilized. Furthermore, additional research into the various forms of video interventions and a thorough investigation into the underlying mechanisms and pathways through which these interventions affect ED is essential. These studies will contribute to refining intervention strategies and improving the overall comprehension of how to efficiently manage and prevent ED in pediatric patients.

This meta-analysis and systematic review reveal a consistent trend toward decreased severity and incidence of ED in children exposed to preoperative video-based interventions. Specifically, in preschool-aged patients, these interventions significantly mitigate the severity of ED. These findings suggest that video-based interventions can positively influence postoperative recovery in young children, highlighting their potential as a beneficial preoperative preparatory tool. This supports the integration of video-based strategies into preoperative protocols to improve recovery outcomes in pediatric surgical patients.

## Conflicts of interest

The authors declare no relevant financial or non-financial interests to disclose.

## Data Availability

The datasets used or analyzed during the current study are available from the corresponding author upon reasonable request.

## Appendix Supplementary materials

Image, application 1
