# Supplementary material for: Effect of video-based interventions on emergence delirium in pediatric patients: a systematic review and meta-analysis of randomized controlled trials
Source: J Pediatr (Rio J). 2024 Sep 6;101(1):21–9. doi: 10.1016/j.jped.2024.06.016 (PMC11763852; doi:10.1016/j.jped.2024.06.016)

**JPED-D-24-00123 – Supplementary Material**

**Effect of Video-Based Interventions on emergence delirium in pediatric patients: a systematic review and meta-analysis of randomized controlled trials**

**Supplemental materials**

**Supplemental Appendix 1, the search strategy**

**PubMed (**[**https://pubmed.ncbi.nlm.nih.gov/**](https://pubmed.ncbi.nlm.nih.gov/)**)**

**1# Delirium**

(((((((((((((((((((Delirium, Emergence[Title/Abstract]) OR (Emergence Agitation[Title/Abstract])) OR (Agitation, Emergence[Title/Abstract])) OR (Agitations, Emergence[Title/Abstract])) OR (Post-Operative Delirium[Title/Abstract])) OR (Delirium, Post-Operative[Title/Abstract])) OR (Post Operative Delirium[Title/Abstract])) OR (Postanesthetic Excitement[Title/Abstract])) OR (Excitement, Postanesthetic[Title/Abstract])) OR (Anesthesia Emergence Delirium[Title/Abstract])) OR (Delirium, Anesthesia Emergence[Title/Abstract])) OR (Emergence Delirium, Anesthesia[Title/Abstract])) OR (Postoperative Delirium[Title/Abstract])) OR (Delirium, Postoperative[Title/Abstract])) OR (Agitated Emergence[Title/Abstract])) OR (Emergence, Agitated[Title/Abstract])) OR (Emergence Excitement[Title/Abstract])) OR (Excitement, Emergence[Title/Abstract])) OR (acute delirium[Title/Abstract])) OR (Emergence Delirium[MeSH Terms]) **5233**

**2# video**

((((((cartoon video[Title/Abstract]) OR (video[Title/Abstract])) OR (videogame[Title/Abstract])) OR (virtual reality[Title/Abstract])) OR (virtual reality video[Title/Abstract])) OR (virtual reality tour[Title/Abstract])) OR (game[Title/Abstract]) **178030**

**3#**

1# AND 2# **44**

**4# voice**

((voice[Title/Abstract]) OR (mother voice[Title/Abstract])) OR (maternal voice[Title/Abstract]) **40651**

**5#**

1# AND 4# **10**

**6#**

3# AND 5# **54**

**Supplemental Figure 1, risk of bias summary.**


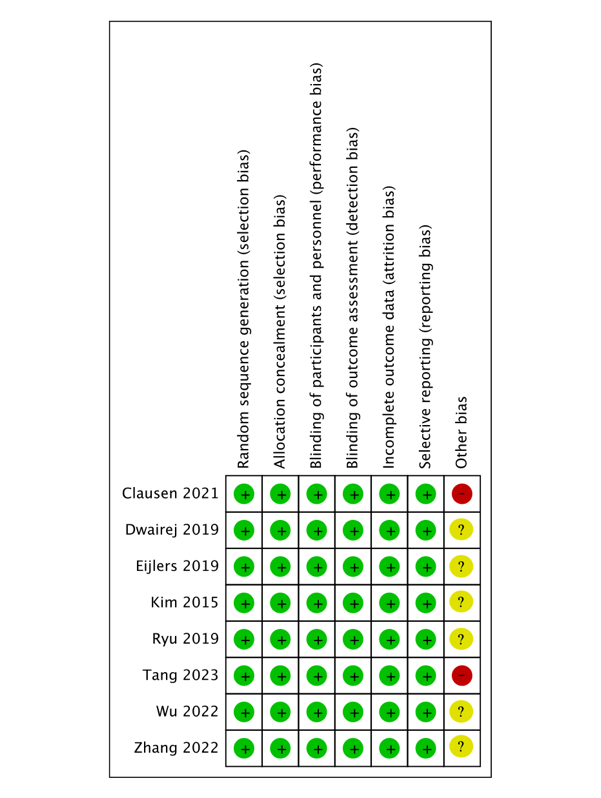

Supplement: Supplementary file 1 [file mmc1.docx]
